# An Account of Two Successful Operations for Restoring a Lost Nose (Lost Noses) from the Integuments of the Forehead, in the Cases of Two Officers in His Majesty's Army : To Which Are Affixed, Historical and Physiological Remarks on the Nasal Operation; Including Descriptions of the Indian and Italian Methods

**Published:** 1817-11

**Authors:** 


### PART II.

### COMPREHENSIVE ANALYTICAL REVIEW OF MEDICAL LITERATURE.

"Tros, tyriusve, nobis nullo discrimine agetur."

*An Account of two successful Operations for restoring a lost Nose (lost Noses) from the Integuments of the Forehead, in the Cases of two Officers in His Majesty's Army: to which are affixed, Historical and Physiological Remarks on the Nasal Operation; including Descriptions of the Indian and Italian Methods.* By J. C. CARPUE, &c. &c. One Vol. Quarto, pp. 104, with Engravings. London, 1816.

IT has long been a disputed point, whether HONOUR holds her principal seat and throne in the most prominent feature of the face, or on a posterior eminence in the vicinity of the sacrum. Certain it is, that a digital salute of the one, or a pedal of the other, "hurts honour more" than wounds of the most noble and vital organs in the body. Independent of all honorary or olfactory feelings, however,

> It must be confessed, that the prejudice goes
> Very much in the favour of wearing a NOSE;

And since both Mars and Venus are making continual depredations on this important feature, it is not to be wondered at, that "*the nasal operation*" should have excited the attention of royalty itself, and called forth *princely* patronage towards the revivor of the far-famed Taliacotian art. We are far from undervaluing the merits of Mr. Carpue's operation; for we have seen too many melancholy instances of those tyrannical dismemberments so common in the East, and which, indeed, gave origin to the Oriental art of nose-mending, not to appreciate the humanity of obliterating so great and so cruel a deformity. We shall, therefore, give a very full analysis of the work before us, because its shape and price must considerably curtail its circulation among the lower orders of the profession.

*Historical and Physiological Remarks.* Peter Branzano, in the year 1442, relates, that one Branca, a Sicilian surgeon, practised a method of repairing ears, lips, and noses. His son Anthony distinguished himself in the same art.

"Orpianus, (says Elysius Calentius, a Neapolitan poet) if you would have your nose restored, come to me. Truly, the thing is wonderful. Branca, a Sicilian, a man of great abilities, has learnt the art of restoring a nose, either by supplying it from the arm of the patient, or by infixing upon the part the nose of a slave."—*Gourmelenu's Chir. Art.* 1580.

Gabriel Barri also speaks of a Vincent Vianeus, of Tropea, who restored defective lips and noses. The art was practised by his sons. Alexander Benedictus, who taught medicine at Padua, in the year 1495, mentions this operation.

"In our times, skilful persons have taught us how to rectify deformities of the nose. Portions of flesh cut from the arm of the patient, formed into the shape of nostrils, and added to the trunk of the nose, are very commonly seen."

Gabriel Fallopius, who died at Padua in 1563, treats of these restorations. Ambrose Paré, in 1561, mentions the circumstance of the Italian surgeons practising these operations; and instances the case of one of the Sancto Thoanos, whose nose was restored.—Andrew Vesalius, a native of Brussels, in 1569, treats at some length of the Taliacotian art, quoting a variety of authorities in support thereof.—Stephen Gournælen, the adversary of Paré, in 1580, repeats the assertion of Calentius, that the nose was sometimes formed or fitted from that of a slave.

Gaspar Taliacozzo, or Taliacotius, was born at Bologna, in the year 1546, and died in the same city in 1599. He filled the chair of anatomy in this city, and enjoyed very high reputation, particularly for his operations on the ears, lips, and nose. It was not till the year 1597, that he printed in folio, at Venice, his work *de Curtorum Chirurgia per Insitionem.* This scarce and singular work is comprised in two books, containing forty-five chapters. Taliacotius, according to the taste of the times, swells out his chapters with all manner of irrelevant matters. In the eleventh, however, he arrives at the subject. In this and the succeeding, the principles of the operation are developed. In the thirteenth, he observes, that the *integuments* form the material from which the deficient parts must be supplied, and not from muscle. In the fourteenth chapter, Taliacotius decides on the places whence the skin should be taken for the nose, lips, or ears. For the two first, the arm above the elbow, and for the last, the skin behind the ears are to be selected. The fifteenth and sixteenth chapters contain directions as to the quantity of skin to be taken, and the manner in which the parts are to be kept in approximation till natural union takes place. The parts are to be united by the interrupted suture. In the eighteenth chapter Taliacotius distinctly denies that the supplementary integuments should ever be taken from a second person. Not that union would not take place, but because impatience and mal-position would be likely to frustrate the object of the operation. The subsequent chapters of the first book are not so important. In the second book, Taliacotius describes the operation, together with the instruments and necessary apparatus. A portion of integument is to be first marked out on the arm; an incision to be made on each side, while the upper and lower ends remain untouched. This portion of integument is next to be dissected from the muscle beneath, and a piece of linen passed under it. This done, the patient is to be kept quiet, and the wound preserved as much as possible from inflammation for some days.

The next step is to detach one end of the graft or flap of integument from the arm, which flap is to be turned so that the natural surface may be outermost. If a nose is to be supplied, that end of the slip nearest to the axilla is to be cut. By means of a dress and apparatus, which are delineated in a wood-cut, the arm and nose are now brought in contact, the surgeon first dissecting away the integuments of the deficient member. The graft being now brought to the nose by lifting the arm, and being found to fit, is to be secured thereto by ligatures. The patient is now bound so that he cannot move, and cloths wetted with white of eggs and rose water, to be applied to the wounded parts. The remainder of the second book describes the manner in which, at the end of twelve days, the arm is to be released, and the graft cut from it; the manner of modelling the septum; the plasters and bandages, &c. &c.

Fienus, a native of Antwerp, bears the strongest testimonies to Taliacotius's cures; the reality of which have sometimes been doubted. He asserts that he was an eye witness of many of them.

Fabricius Hildanus bears on the same point. John

Baptist Cortesi, born at Bologna 1554, died in 1636, describes the method of Taliacotius, and asserts that he himself carried it into practice. Anthony Molinetti, a Venetian, who filled the chairs of anatomy and surgery at Padua, in his Dissertations on the Senses and their Organs, praises the Taliacotian method; and asserts, in proof of its practicability, that his father restored, in the year 1625, the nose of a Noble Polonese.

With these notices, the history of the Taliacotian operation may be considered as near its close. It now became neglected; and Heister appears to discredit it.

"What is there proposed (by Taliacotius) is, for want of later experiments and observations, judged to be impracticable and without foundation, by our modern surgery." *English Edition*, 1768, *chap.* 73.

M. Eloy, in his Dictionnaire Historique (1778), expresses himself to the same effect, as does M. Petit Radel, in the Encyclopedie Methodique, 1792. In England, Wiseman does not even mention the operation; and Mr. Carpue affirms, that John Hunter entirely mistook the whole history of the Taliacotian method. Dr. Thomson, in his Lectures on Inflammation, appears to think that the pain and discomfort of the operation (referring to the Italian method of the arm), exceed the value of the benefit it promises.

In accounting for the neglect of the operation, Mr. C. adduces the ridicule which was attached to it at and since the time of Charles the Second; as it was then ranked with the exploits of Jack the Giant Killer, and spoken of to amuse children. It is to the superstitious stories of sympathetic influence, and not to the works of Taliacotius, that Butler refers, in his well-known lines on

"—— supplemental noses, which

Would last as long as parent-breech;

But when the date of Nock was out,

Off dropp'd the *sympathetic* snout."

*Indian Method.*—When the nasal operation had become forgotten or despised in the West of Europe, it was once more heard of in England, from a quarter whence mankind will yet, perhaps, derive many lights, as well in science as in learning and in arts. The Gentleman's Magazine for 1794, contains a communication, of which the following is an outline. Cowasjee, a bullock-driver in the English army, was made prisoner by Tippoo in 1792, who cut off his nose and one of his hands. In this state he joined the Bombay army near Seringapatam. For twelve months he was without a nose, when a Mahratta surgeon supplied him with one. The operation is not unusual in India, and has been practised time immemorial. Mr. Thomas Cruso, and Mr. James Findlay, saw it performed thus:

"A thin plate of wax is fitted to the stump of the nose, so as to make a nose of good appearance; it is then flattened, and laid on the forehead. A line is then drawn round the wax, which is of no farther use; and the operator then dissects off as much skin as it covered, leaving undivided a small slip between the eyes. This slip preserves the circulation till an union has taken place between the old and new parts. The cicatrix of the stump of the nose is next pared off; and immediately behind this raw part, an incision is made through the skin, which passes round both alæ, and goes along the upper lip. The skin is now brought down from the forehead; and being twisted half round, its edge is inserted into this incision, so that a nose is formed with a double hold, above, and with its alæ and septum below, fixed in the incision. A little terra japonica is softened with water, and, being spread on slips of cloth, five or six of these are placed over each other, to secure the joining. No other dressing than this cement is used for three or four days: it is then removed, and cloths dipped in a kind of butter, are supplied. The connecting slip of skin is divided about the twenty-fifth day; when a little more dissecting is necessary to improve the appearance of the new nose. On the tenth day after the operation, bits of soft cloth are put into the nostrils to keep them open. This operation is always successful."

Mr. Pennant, in his view of Hindostan, gives also a description of this operation. Mr. C. made enquiries of Gentlemen who had resided in India, who confirmed these accounts. Mr. James Stuart Hall had seen the operation performed, and said it was tedious. Dr. Barry of the India service, had also seen it done. It occupied an hour and a half, and was performed with an old razor; tow was introduced to support the nose, but no attempt to form nostrils, by adding a septum, was made. Mr. Lucas, an English surgeon, copied the Indian method there, and was often successful.

"A due attention to the little evidence within our reach, will go far to fix us in the opinion, that the nasal operation is of the highest antiquity in the warm climates of the East; that its practice, in all the southern parts of Asia, from India to Persia and Arabia, has existed from very early times; that Greece shared it with Asia; that it afterwards passed into Calabria, and from Calabria into other parts of Italy, and that in Italy it languished, and died; partly through its approach to severer climates, partly, perhaps, through the practice of a more complicated, painful, and tedious method; partly through the low state of surgery in Europe; and partly through the senseless incredibility, and ridiculous misconceptions, arising out of that low state of surgery, with which it was met both by practitioners and people." p. 44.

Traces of this Art may be gleaned from the works of Celsus, Galen, Paulus Ægineta, &c. who, in all probability received them from the eastern world.

Under the head of physiological remarks, Mr. Carpue alludes to the well-known instances of divided parts re-uniting, as published by Dr. Balfour and others. Dr. B's cases have received every publicity, but we shall glance at some of the similar cases collected by Mr. C.

Bossu, a surgeon of Arras, states the case of a thumb thus:

"A boy, in shaping a wedge of wood, amputated obliquely the third phalanx of the thumb of the left hand, a little above the nail, in such a manner as to lay open the articulation on the lateral and internal part. He came to me immediately, and while the wound was still bleeding. I took the thumb, which he had in his pocket, covered with dirt and crumbs of bread, washed it in warm wine, re-applied it, and supported it with proper dressings." p. 59. *Journal de Medecine.*

Mr. Abernethy informed our author, that an instance of the extremity of a finger quite removed, and again re-uniting, occurred not long ago at St. Bartholomew's hospital.

We shall pass over a great many pages containing observations, on adhesions, &c. in order that we may give a fuller analysis of the two cases whence originated the volume before us.

Case 1. In September, 1814, Mr. C. was applied to by an army officer, whose nose was in a mutilated state, and who requested to have the nasal operation performed on him. The patient had gone to Egypt in 1801, where he contracted a liver complaint; the usual remedy in such a case, mercury, was employed in Malta, in Egypt, and subsequently in Ireland. On his return to London, he consulted Mr. Heaviside, who advised him to discontinue the use of mercury; being decidedly of opinion that the sore throat he complained of was *mercurial*, and not *venereal*. The gentlemen, to whose care he committed his health, were unfortunately of a contrary opinion; and hence, the mercury was persevered in. Shortly after, his nose became affected, which confirmed the practitioners in their belief; the mercury, upon this, was increased. The septum of the nose having begun to slough, and the constitution of the patient appearing to be greatly injured, the mercury was at length laid aside. Thus relieved from the occasion of his sufferings, he gradually recovered his health; but with the loss of the septum, the anterior part of the cartilage, and the whole front of the nose, except a small portion of the alæ.

Mr. Carpue was most anxious to determine whether this was a case in which success would in all probability follow; considering, that if success followed the attempt, he would introduce into the English practice an operation, which he had for many years recommended to his pupils, but that a failure, from whatever cause would fix an indelible stigma on the operation, as fruitless and visionary. In this case the parts had sloughed from disease; in India they had been divided by art. The constitution too of the patient had been impaired by the liver complaint, and improper treatment, which, independent of the difference of climate, rendered the prospects in this case by no means favourable. Under pretext, therefore, of preparing for the operation, he made incisions near the remains of the alæ. Being satisfied of complete success by the healing of the wound, he commenced a series of operations on the dead subject, before a number of medical friends; and on the 23d of October, proceeded to perform the operation. It should be observed, that this was rendered more difficult from the unusual lowness of the patient's forehead; Mr. C. fearing that the hair would again grow after its extirpation, and prevent adhesion about the new alæ nasi.

Having ascertained the size of the graft required by means of a wax model, which he flattened and laid on the patient's forehead, Mr. Carpue drew the outline with red paint; as also on the sides, and beneath for the septum where incisions were to be made. The patient then laid himself on his back on a table, refusing to be held; saying, "I hope I shall behave like a man." Nor did he make the smallest complaint through the operation.

Mr. C. now dissected out a sufficient quantity of the face to receive the graft from the forehead. He then began that part of the operation which belongs to the forehead, by dissecting the integuments along the lines he had drawn, merely leaving the pericranium. The angular artery bled freely; there was, however, no occasion for tying it. The part which hung down became of a purple colour, and the patient complained that his forehead felt extremely cold. The application of warm sponges afforded great relief, and was continued during the remainder of the operation. He next made a turn of the dissected parts, and introduced the septum into the incision of the upper lip, where he confined it by ligature. Two ligatures on the left, and two on the right, brought the integuments exactly into contact; lint was then introduced to distend the nostrils, and straps of adhesive plaster preserved every part in its proper situation. The last step was to bring the edges of the integuments on the forehead, and between the eye-brows, as near together as possible, and to keep them so by means of adhesive plaster. The dissection occupied nine minutes, and the ligatures six. After this the application of the necessary bandages, changing the patient's linen, and placing him in bed, consumed twenty-two minutes more. Short, however, as was the time, Mr. C. expresses his surprize at the fortitude with which his patient went through this operation, particularly as there were in his case no prior examples of success in England, to support his confidence.

The patient, being put to bed, enjoyed some sleep. In the night there was a trifling hæmorrhage. Perfectly quiet the next day; pulse as before the operation. Much inclination for food, but allowed only jellies and barley-water.

On the third day, Mr. C. took off the dressings. It is easier to conceive than to describe his high satisfaction, on finding that adhesion, agreeably with his most sanguine hopes, had taken place in every part, and that the nose was of the same colour as the face. It was, however perfectly flat, and rose and fell with every inspiration and expiration. To remedy this unsightly state of the nose, Mr. C. had thoughts of procuring the air-bladder of a fish, which he proposed to introduce into the nose, and then inflate, with the design of raising the point of the nose. His apprehsions however in this, as in other parts of the cure, were groundless. Nature worked with him, and raised the nose by her own means.

By the the sixth day after the operation, all the ligatures were removed; granulations were forming freely on the forehead. On the ninth day, through carelessness in mastication, a small portion of the newly united parts were divided from the old on the left side. The fissure was, however, agaiu made to unite. About the twelfth day the nose became very large; so much so that Professor Assalini, who saw it at that period, advised a part of it to be removed. Mr. C. however suggested, that it was merely œdematous; and that absorbent vessels would form in time, as well as arteries; his opinion proved to be correct, for in a month after, absorption had removed the deformity. Four months after the operation, Mr. Carpue dissected the integuments on the bridge of the nose, which he united from the turn which had disappeared, and confined them by ligature; being of opinion, that if he followed, in this cold climate, that part of the Indian practice, which consists in removing the turn by which circulation is carried on from the parent parts, the nose would slough; as seems to have been the case in the story related by Van Helmont, and others. In no other particular of importance did he depart from the Indian method.

After the œdema had subsided, the nose was left very flat; granulations, however, formed, and the nose experienced a healthy enlargement. In the present state of the nose, though there is neither bony nor cartilaginous septum, yet the anterior part is solid, and has every appearance of a natural nose. The nostrils are gradually growing bigger, and the secretion takes place as usual. The forehead was healed in three months, and the cicatrix diminished to a very trifling size.

Case 2. At the battle of Albuera, May 16, 1810, Lieutenant Latham, of the 3d foot, in a desperate struggle for the preservation of the colours of his regiment against a body of Polish lancers, lost an arm by a sabre cut, and received five wounds, one of which took off part of his cheek and nose. He was afterwards found, in a state of insensibility, on the field, with the colour under his body. This transaction having recommended him to the notice of the Prince Regent, his Royal Highness was pleased to place him under Mr. Carpue's care, requesting that he would give his unfortunate case his utmost attention.

In the state in which the nose was left by the sabre of the enemy, all the interior was exposed. Exclusively of the disagreeable appearance produced, Lieut. Latham suffered from repeated colds and inflammations. The right side of the alæ remaining entire, the patient preferred a cicatrix in the middle of the nose to an amputation of the sound part. Mr. Astley Cooper, Mr. Sawrey, and Mr. Anderson, surgeon to the 3d regiment, agreed in opinion, that the integuments taken from the forehead would unite with those which covered the side of the nose.

An incision was made on the forehead, as in the former case, as also similar dissections. On making the dissection on the forehead, there was a very considerable hæmorrhage; so much so, that an artery was obliged to be secured. When the part hung down, it did not appear purple, as in Case 1; and an artery from it bled as freely as the temporal artery when opened. This, however, subsided; and there was no occasion for a ligature. The parts were brought into contact, as in Case 1, by means of five ligatures; the only difference was, that the right side of the integuments, instead of being received into an excavation, were brought into exact contact; and that the lip was not divided, but the lower part of the septum dissected away, and the new part brought into contact, and detained there by ligature. Adhesive plaster was applied to the forehead as before, and the usual dressings.

The patient had some sleep the first night, but was feverish: more hæmorrhage than in Case 1. This case proceeded in the same manner as the first, with very little variation, except that there was much inflammation, and suppuration from the internal parts. The parts became œdematous about the sixth day: the œdema, however, decreased daily, pressure appearing advantageous in this instance. In six weeks the forehead was completely healed, and the cicatrix was very small.

On the 7th of October, Mr. C. performed the second part of the operation, which consisted in making a longitudinal incision on the top of the nose, dissecting away a considerable portion of the under surface of the new nose, and making an incision corresponding to that of the old nose. He then brought the two incisions in the old and new noses exactly in contact, uniting them by two silver pins, and the twisted suture. Two days after, a perfect adhesion having taken place, he withdrew the pins, and applied adhesive plaster. In ten days, the parts were perfectly recovered. A dissection of the new nostril remains to be made.

We have thus given a very full analysis of the nasal operation, as well as its history; leaving the reader to form his own estimate of the importance of this branch of surgery. For our own parts, and from what we have heard of the *present* appearance of some of these supplementary noses, we are not disposed to augur very favourably of the operation in *this* country; although we give Mr. Carpue every credit for his zeal and intrepidity in the enterprize.

The Engravings are very illustrative of the steps of the operation, and the other subjects they are designed to represent.

In order not to break the chain of narrative, we have extracted the following Case, and detached it from its central situation in Mr. C's work.

### *Case of wounded Carotid Artery.*

"On the 3d of October, 1803, Mr. G. a shoemaker, residing in Little Coram Street, Tavistock Square, being engaged in a quarrel with his wife, went behind her, and with a square shoemaker's knife stabbed her in the side of the neck. A violent hæmorrhage followed; but a female lodger in the house, who had formerly been an hospital nurse, happened to pass the room-door at the moment, and immediately flew to the woman's assistance. Having a cloth already in her hand, she applied it to the wound, and kept it there with the strongest pressure. Dr. Cairns, formerly a surgeon in the navy, and practising as a physician at the Cape of Good Hope, instantly attended. This gentleman used every effort to tie the jugular vein and left common carotid artery, both of which were divided; but the hæmorrhage was so considerable, that this was impracticable. In this state of the patient, he desired my assistance. On hearing his account, I requested him to remove the pressure, in order to my seeing the part; but he said, that this was impossible, as the patient would immediately bleed to death, and assured me that the use of the tenaculum and forceps was impracticable.

"In this extremity, I proposed to him to pass a ligature round my wrist, to be used as hereafter described; and this done, to take off the pressure, and admit my thumb and finger into the wound. The hæmorrhage, as he had forewarned me, was truly dreadful; but I succeeded in laying hold on every part within the wound, theca, artery, vein, and nerves, down to the vertebræ. I now desired him to slip the ligature over my fingers, and make it tight on the parts which I held; which being done, the hæmorrhage completely ceased. At the time the ligature was tied, the left eye protruded dreadfully from its orbit. Mr. Tweedie, of Southampton Row, was present, and assisted in tying the artery." p. 63.

"The woman was kept in the same position, and forbidden to eat or drink. Sir Richard Ford attended, from Bow Street, to take her deposition; but I told him, that if she lived, which I much doubted, it would be from her being kept perfectly quiet. Mr. Allcock, my pupil, since deceased, remained with her. So little hope had I of her recovery, that I informed Mr. Allcock that I would return in two hours, when I supposed she would be dead; that we would dissect the parts, when I apprehended we should find, that we had not only tied the jugular vein and common carotid, but included the par vagum and great intercostal nerve. On my return, however, to my equal surprise and gratification, she was still living. Her pulse was very low, and the right side of her head was very cold: she had some sleep, and did not seem to be in much pain. Sixteen hours after tying the artery, I allowed her a small quantity of cold barley water. I kept strictly on the antiphlogistic plan for eight days, scarcely allowing her any food, and none which required mastication; when the ligatures came away without the least hæmorrhage and the parts gradually healed from the bottom. The woman's age was about fifty-five; and, though neither sickly nor emaciated, she was of spare habit."

---

